# Phosphorus leaching from loamy sand and clay loam topsoils after application of pig slurry

**DOI:** 10.1186/2193-1801-1-53

**Published:** 2012-11-28

**Authors:** Jian Liu, Helena Aronsson, Lars Bergström, Andrew Sharpley

**Affiliations:** 1 Department of Soil and Environment, Swedish University of Agricultural Sciences, Box 7014, 75651 Uppsala, Sweden; 2 Department of Crop, Soil and Environmental Sciences, University of Arkansas, Fayetteville, Arkansas 72701 USA

**Keywords:** Intact topsoil column, Manure management, Mineral fertilizer, Phosphorus leaching, Phosphorus loss, Pig slurry, Slurry incorporation

## Abstract

Appropriate management of animal waste is essential for guaranteeing good water quality. A laboratory leaching study with intact soil columns was performed to investigate the risk of phosphorus (P) leaching from a clay loam and a loamy sand. The columns (0.2 m deep) were irrigated before and after application of pig slurry on the surface or after incorporation, or application of mineral P, each at a rate of 30 kg P ha^-1^. The two soils had different initial P contents (i.e. the ammonium lactate-extractable P was 65 and 142 mg kg^-1^ for the clay loam and loamy sand, respectively), but had similar P sorption characteristics (P sorption index 3.0) and degree of P saturation (17-21%). Concentrations of dissolved reactive P (DRP) and total P (TP) before P application were significantly higher in leachate from the loamy sand (TP 0.21 mg L^-1^) than from the clay loam (TP 0.13 mg L^-1^), but only increased significantly after P application to the clay loam. The highest concentrations were found when slurry was surface-applied (DRP 1.77 mg L^-1^), while incorporation decreased the DRP concentration by 64% in the clay loam. Thus moderate slurry application to a sandy soil with low P saturation did not pose a major risk of P leaching. However, application of P increased the risk of P leaching from the clay loam, irrespective of application method and despite low P saturation. The results show the importance of considering soil texture and structure in addition to soil chemical characteristics in risk assessments of P leaching. Structured soils such as the clay loam used in this study are high risk soils and application of P to bare soil during wet periods, e.g. in autumn or spring, should be followed by incorporation or avoided completely.

## Introduction

Reduction of phosphorus (P) losses from arable land is of major concern within the work to reduce eutrophication of waters, as for example the Baltic Sea in northern Europe (Boesch et al. 2006). The processes behind P losses are complex and influenced by natural factors, such as soil properties, topography and weather conditions, as well as by different management practices in the field. These practices include for example crops, cropping sequences, fertilizer applications (Sharpley et al. 2004; Sims et al. 1998) and soil tillage (Ulén et al. 2010).

Phosphorus losses occur with large variations in both time and space (Djodjic et al. 2000; de Jonge et al. 2004), which drives the need for development of index-based model tools for identification of critical source areas and mitigation options (Heckrath et al. 2008; Sharpley et al. 2003). For development of such tools, knowledge is needed about causes of losses and pathways of P transport to identify the areas and time periods susceptible for P losses, not only at the farm level but especially within individual fields. Moreover, knowledge is also needed about how different management practices interact and affect mobilization or retention of P in the soil to enable site-specific targeted design of measures. This site-specific approach also implies that low-risk areas for P losses must be considered if measures are to be cost-efficient (Johansson and Randall 2003). For some areas no additional efforts might be needed to keep P losses small.

For areas with intensive livestock production, appropriate manure management is one of the most important issues with respect to P leaching losses (Smith et al. 1998). The long-term build-up of soil P content and the associated risk of increased P leaching has been investigated and verified in different types of studies (Heckrath et al. 1995; Hooda et al. 2000; Börling et al. 2004). However, in studies of whole soil profiles this relationship is sometimes strong but often weak or even absent due to different P sorption characteristics of the subsoil (Siemens et al. 2004; Djodjic et al. 2004; Liu et al. 2012a). The presence of preferential flow paths may have a large influence on the transport of water which is important to evaluating the risk of P losses (Bergström and Shirmohammadi 1999). Single applications of mineral or organic fertilizers may also constitute a direct risk of P losses, especially if applied in large amounts and/or during conditions with risk of surface runoff or leaching. This risk is mainly attributed to structured soils as clay and clay loams, where macropores may provide rapid transport of solutes and particles (Jarvis 2007). In contrast, for coarse-textured soils without macropores, the direct risk of P leaching losses after application of P is generally low due to adsorption of P (van Es et al. 2004; Glæsner et al. 2011; Sørensen and Rubæk 2012).

There may be possibilities to reduce P losses from soils with macropore flow pathways. Stabilization of the macropore system seems to be a factor which enhances the risk of losses due to transport of dissolved P (Shipitalo et al. 2000). No-till systems and permanent grassland have shown increased P leaching due to the build-up of continuous macropores (Culley et al. 1983), which can be broken by use of tillage (Geohring et al. 2001). However, soil tillage is not always the solution, which was found by Schelde et al. (2006), who measured increased transport of particle-bound P after plowing grassland, as the particles in the plow layer were loosened by plowing (Øygarden et al. 1997). Siemens et al. (2008) also suggested that mechanical disturbance by plowing in combination with an existing macropore system may result in enhanced leaching of colloidal P. To avoid rapid transport of dissolved P in macropores, the amount of dissolved P should be low during periods when macropore flow may occur, and/or the contact between the soil and dissolved P should be increased. This is to be an important measure when P is applied before rain events both for mineral and organic fertilizers. For example, Djodjic et al. (2002) found significantly decreased P leaching during one of three years after application of mineral P which was mixed with the topsoil. Glæsner et al. (2011), who compared surface application and injection of dairy slurry, concluded that P leaching from irrigated topsoil columns of sandy loam and loam decreased by about half when the slurry was injected. Injection of slurry may certainly also affect runoff losses of P, which was shown by Uusi-Kämppä and Heinonen-Tanski (2008) who found that P runoff losses were reduced by about 80% compared with broadcasting, when slurry was applied to perennial grassland. Also Kleinman et al. (2002) observed considerably reduced risk of P runoff losses for different soils, when manure was mixed into the soil.

Although not optimal from a nutrient management perspective, there is a need on many farms, for practical reasons, to apply manure during periods of increased rainfall-runoff potential, as during spring and autumn in regions with cold and wet climates.

In this study we investigated two soils from an area with intensive livestock production in southwest Sweden. One was presumably a low-risk sandy soil without macropores and with low P saturation. The other one was a clay loam which could possibly be a high-risk soil according to studies on similar soils. We used intact topsoil columns of these two soils for studies under intensive simulated rainfall conditions. Mineral P or pig slurry, with and without incorporation, was applied. The main objective was to investigate the hypotheses that 1) on the sandy soil P could be applied at moderate rates without increased risk of P leaching, and 2) that a possible risk of P leaching from the clay loam could be satisfactorily reduced by incorporation of the applied slurry. Moreover, an objective was to compare P leaching from different P sources, i.e. mineral fertilizer and pig slurry.

## Materials and methods

### Soil column sampling and preparation

A total of 16 replicate intact topsoil columns (0.2 m deep) were collected from each of the two experimental fields at the Lilla Böslid experimental station in the coastal region of southwest Sweden (56^o^35′N, 12^o^56′E) with relatively high precipitation (803 mm yr^-1^, Halmstad 1961–1990). These two fields are situated a few hundred meters apart on a loamy sand and clay loam soil, respectively, in which separately tile-drained experimental plots for nutrient leaching studies were installed in 2002 and 2008. Selected soil physical and chemical properties of the topsoils are shown in Table 1. The loamy sand (P-AL: 142 mg kg^-1^) has a higher P content in the topsoil than the clay loam (P-AL: 65 mg kg^-1^); while the two soils have the same P sorption capacity expressed as P sorption index (PSI) (Börling et al. 2001). A more detailed description of the field with loamy sand was presented by Aronsson et al. (2011) and Liu et al. (2012b).

**Table 1:** **Selected soil physical and chemical properties of the topsoil (0–0.2 m), with some values for the subsoil (0.2-1 m) included in brackets. Some values were presented as Mean ± S.D. (n=4)**

Soil properties	Clay loam	Loamy sand
Physical properties		
Soil texture (%)		
Clay (<0.002 mm)	29 (39)	7 (1)
Silt (0.002-0.0625 mm)	43 (43)	5 (0)
Sand (0.0625-2 mm)	28 (18)	88 (99)
Saturated hydraulic conductivity (cm h^-1^)	1-350 (2–2000)^#^	0.2-8 (0.1-12)
Chemical properties		
pH (H_2_O)	6.5	6.0
Total C (%)	2.2	2.5
P-AL (mg kg^-1^)	65 ± 17	142 ± 9
Fe-AL (mg kg^-1^)	270 ± 20	140 ± 10
Al-AL (mg kg^-1^)	210 ± 30	540 ± 50
DPS-AL (%)	16.9 ± 4.2	20.7 ± 1.6
PSI	3.0 ± 0.1	3.0 ± 0.4

#: Hydraulic conductivity of the clay loam was estimated according to Karlsson and Håkansson (1983).

The soil columns, encased in 20-cm long and 18.8-cm inner diameter PVC pipes were collected after harvest in September 2009, by use of hydraulic pressure. The collection points of the replicate columns were evenly distributed along one diagonal of each field. The columns were sealed and stored in a cool room at 4°C until the leaching experiment started in the laboratory. The procedure of collecting and preparing columns was described in detail by Liu et al. (2012b) and differed only with respect to how the bottoms were prepared. In this study, the knife was kept vertical to make sure soil aggregates were broken at the weakest part when cutting off the excessive soil at the bottom of clay loam columns.

### Leaching experiment

A leaching experiment was conducted in two sequences, before and after application of pig slurry or mineral P fertilizer. Simulated rainfall was applied to each column for four events during each sequence by equipment described by Liu et al. (2012b). The rainfall was simulated with tap water with chemical compositions: pH = 8.2, EC = 45 mS m^-1^, 106 mg HCO_3_ L^-1^, 35 mg Ca L^-1^, 15 mg Mg L^-1^, 20 mg Na L^-1^, 40 mg SO_4_ L^-1^, 40 mg Cl L^-1^, 0.02 mg total P L^-1^ and 1.6 mg total N L^-1^. The simulator was calibrated to have a rotation of 26 seconds of rain under a pressure of 50 kPa, and 37 seconds of no rain, which gave a rain intensity of 10 mm hr^-1^. Each rainfall event lasted for two hours, giving a total rainfall of 20 mm event^-1^. The total amount of water applied during all four events (80 mm) corresponded to approximately one pore volume. Leachate samples were collected from each gravity-drained column after every rainfall event. Two rainfall events were simulated on one day, with an interval of 6–16 hours between two events. This time interval was enough for most of the applied water to drain out and be collected just before the following rainfall simulation started.

After the first sequence of four leaching events, the columns were naturally drained for 60 hours before P was applied. There were three experimental treatments with P application and one control without for each site, each with four replicate soil columns. Two treatments received pig slurry which was either surface applied (SP-Surf) or incorporated (SP-Incor). One treatment received mineral P fertilizer (superphosphate Ca(H_2_PO_4_)_2_). The application rate for pig slurry and mineral fertilizer was 30 kg P ha^-1^. This application rate of slurry is slightly higher than the mean annual amount of manure that can be applied to agricultural soils in Sweden (22 kg P ha^-1^; i.e. <110 kg P ha^-1^ with animal manure over a 5-year period for the entire farm area) (SBA 2010). Thus, the P application rate selected represents a common practice for autumn application of manure. The slurry, collected from a farm with fattening pigs and manure storage under cover, had a dry matter content of 7.3% and a total P (TP) content of 1.7% of dry matter (Table 2). Of the TP in slurry, 49.5% was extractable by NH_4_Cl, which is a loosely bound fraction of P (Hieltjes and Liklema 1980) and thus easily available for crop uptake and leaching. For incorporation of the slurry, the upper 1-cm soil layer was removed and mixed with the slurry before being returned to the soil surface. In the mineral P treatment (MinP), the fertilizer granules, in which 95% of TP was water soluble, were evenly distributed on the soil surface. The columns were then stored at 4°C for two weeks before the second rainfall simulation sequence started, to establish equilibrium between soil and incorporated slurry P. The four treatments are summarized in Table 3.

**Table 2:** **Selected properties of superphosphate and pig slurry applied to the soil columns, and different P fractions of total P in the slurry**

Properties of P sources	Values
Superphosphate	
Water soluble P, % of total P	95
Pig slurry	
Dry matter content^#^, %	7.3
Total C, %	31
Total N, %	4.0
Total P, %	1.7
P fractions of total P (%)	
NH_4_Cl-P	49.5
Bicarbonate/dithionite-P	0.5
NaOH-P	0.7
HCl-P	0.4
NaOH org-P	1.1
Rest-P	47.7

#: Dry matter content is based on fresh weight and the other properties on dry matter.

**Table 3:** **A summary of the four experimental treatments in this study**

Treatment	Source of P	Application method	Applied total P (kg ha^-1^)
Control	-	-	0
SP-Surf	Pig slurry	Evenly applied on the soil surface	30
SP-Incor	Pig slurry	Incorporated to the upper 1-cm soil layer	30
MinP	Mineral fertilizer	Evenly applied on the soil surface	30

### Analysis of soil, slurry and water samples

A soil sample, taken at 20-cm depth when preparing the bottom of each column, was dried, ground and sieved. Four samples from each site were randomly selected for chemical analysis. Phosphorus, Al, and Fe in the soil samples were extracted with the ammonium lactate method (Egnér et al. 1960) and analyzed with Inductively Coupled Plasma (ICP) spectrometry (Perkin Elmer, Wellesley, MA) to determine the content of easily available elements (P-AL, Al-AL, and Fe-AL). The concentration of P was analyzed after centrifuging and filtering (0.2 μm) the extracts according to colorimetric methods issued by the International Standard Organization (ISO 15681–1 2003) and values were converted to mg kg^-1^ soil by using measured dry bulk densities. The degree of P saturation (DPS-AL) value (%) was calculated as the ratio between P-AL and Fe-AL + Al-AL, expressed on a molar basis (Ulén 2006). The PSI was analyzed according to Börling et al. (2001) using the method of a single addition of 19.4 mmol P kg^-1^ soil and being calculated as X/log C, where X is the amount of P sorbed by the soil (in mmol kg^-1^ soil) at equilibrium and C is the equilibrium P concentration in the solution (in mmol L^-1^).

The content of TP in slurry and different fractions of P were determined by the Erken Laboratory, Uppsala University (Erken Laboratory 2012), and the data is presented in Table 2. The method used was based on the two methods developed for sediments by Hieltjes and Liklema (1980) and Psenner (1988).

Leachate from each column was collected and weighed in individual glass bottles after each simulated rainfall event. It was analyzed for TP and dissolved reactive P (DRP) according to the colorimetric methods issued by the International Standard Organization (ISO 15681–1^2003^). Total P concentrations were determined on unfiltered samples after oxidation and DRP on filtered samples (0.2 μm) without oxidation. The concentration of other-P, which includes mainly particulate-P (PP), colloidal P and dissolved organic P (DOP), was defined as the difference between TP and DRP concentrations.

### Statistical analysis

The SAS program (Version 9.2) was used for statistical analysis. Differences in leachate amount and Log-transformed concentrations of various P forms and P leaching losses were analyzed with the Mixed Model for repeated measurements (Littell et al. 2006), where a “repeated” statement was used to realize the repeated irrigations on the same column. A significance level of α=0.05 was used throughout this study, unless noted otherwise. Three of the clay loam columns were excluded in the analysis due to severe water ponding and no leachate collected (no infiltration), i.e. one replicate of the control treatment throughout the experiment, and one replicate of the MinP and SP-Incor treatment, respectively, during the rainfall events after P applications.

## Results

### Leachate, concentrations and leaching of P under baseline conditions

In general, the loamy sand had higher amounts of leachate and P leaching than the clay loam before P applications (Table 4). With an input of 20 mm simulated rainfall at each event, on average, 18.1 ± 3.1 mm leachate was collected from the columns of the loamy sand, and 16.4 ± 3.1 mm from the clay loam. The difference was slightly significant (*p* = 0.03). The concentrations of TP from the loamy sand columns (0.21 ± 0.07 mg L^-1^) were significantly higher than those from the clay loam (0.13 ± 0.08 mg L^-1^). The leaching loads of TP followed the same trend as the concentrations. The difference was due to different concentrations of DRP in leachate from the two soils, since the other-P concentrations were nearly the same. DRP accounted for 67% of the concentrations of TP in leachate from the loamy sand, while only 38% from the clay loam.

**Table 4:** **Mean values of leachate amounts, concentrations and leaching loads of different forms of P for the four leaching events before P applications**

Soil	Leachate mm	Total P		DRP		Other-P	
		Conc., mg L^-1^	Load, kg ha^-1^	Conc., mg L^-1^	Load, kg ha^-1^	Conc., mg L^-1^	Load, kg ha^-1^
Clay loam	16.4 a	0.13 a	0.020 a	0.05 a	0.007 a	0.08 a	0.013 a
Loamy sand	18.1 b	0.21 b	0.038 b	0.14 b	0.026 b	0.07 a	0.013 a

Small letters (a, b) indicate significant differences within each table column (α=0.05; n=15 for clay loam; n=16 for loamy sand).

### Leachate, concentrations and leaching of P after P applications

After P applications, an average of 17.4 ± 3.3 mm of leachate was collected from the clay loam and 17.1 ± 2.0 mm from the loamy sand after each irrigation event, without significant differences between treatments (Table 5). For most treatments of both soils, leachate amounts did not significantly differ from before application, with exception of the loamy sand SP-Incor treatment, which was significantly lower (*p* = 0.03; Table 6).

**Table 5:** **Mean values of leachate amounts, concentrations and leaching loads of different forms of P for the four leaching events after slurry or mineral P application to the soil columns**

Soil	Treatment	Leachate mm	Total P		DRP		Other-P	
			Conc., mg L^-1^	Load, kg ha^-1^	Conc., mg L^-1^	Load, kg ha^-1^	Conc., mg L^-1^	Load, kg ha^-1^
Clay loam	Control	17.0 a	0.15 a	0.024 a	0.02 a	0.003 a	0.13 c	0.021 b
	SP-Surf	17.7 a	2.76 c	0.482 c	1.77 d	0.304 d	0.99 e	0.178 d
	SP-Incor	17.5 a	1.39 b	0.238 b	0.64 c	0.110 c	0.74 e	0.128 d
	MinP	17.5 a	1.50 b	0.231 b	1.15 cd	0.174 cd	0.35 d	0.057 c
Loamy sand	Control	18.7 a	0.23 a	0.044 a	0.17 b	0.032 b	0.06 ab	0.011 ab
	SP-Surf	17.9 a	0.17 a	0.031 a	0.11 b	0.019 b	0.07 ab	0.012 ab
	SP-Incor	15.7 a	0.20 a	0.032 a	0.10 b	0.016 b	0.11 bc	0.016 b
	MinP	16.2 a	0.18 a	0.029 a	0.13 b	0.021 b	0.05 a	0.008 a

Small letters (a, b, c, d, e) indicate significant differences within each table column (α=0.05; n=3 for the control, SP-Incor and MinP treatments of the clay loam, respectively; n=4 for all the other treatments).

**Table 6:** **Increase in leachate amounts, concentrations and leaching loads of different forms of P after slurry or mineral P application to the soil columns**

Soil	Treatment	Leachate mm	Total P		DRP		Other-P	
			Conc., mg L^-1^	Load, kg ha^-1^	Conc., mg L^-1^	Load, kg ha^-1^	Conc., mg L^-1^	Load, kg ha^-1^
Clay loam	Control	0.2	0.00	−0.001	−0.01	−0.002	0.01	0.002
	SP-Surf	0.4	2.65***	0.461***	1.74***	0.298***	0.91***	0.163***
	SP-Incor	1.6	1.23***	0.214***	0.56***	0.096***	0.68***	0.118***
	MinP	2.1	1.42***	0.219***	1.13***	0.171***	0.29***	0.048***
Loamy sand	Control	1.2	−0.04	−0.004	−0.02	−0.001	−0.02	−0.003
	SP-Surf	0.7	−0.03	−0.004	−0.03	−0.004	0.00	0.001*
	SP-Incor	−3.5*	0.03	−0.002	−0.01	−0.006	0.04	0.004*
	MinP	−2.3	−0.02	−0.009	0.00	−0.004	−0.02	−0.005

Symbol (*) indicate significant differences between values after P application and before (*: α=0.05; ***: α=0.001; n=3 for the control of the clay loam throughout the experiment, and for the SP-Incor and MinP treatments of the clay loam after the laboratory P application, respectively; n=4 for all the other treatments).

Overall, concentrations of P in the leachate and leaching loads of P from the clay loam significantly increased in all three treatments with either mineral P or pig slurry compared with the control (Table 5) and to before P application (Table 6). The concentrations were also significantly higher than those from the columns with loamy sand after P applications. Surface-applied pig slurry to the columns (SP-Surf) had a mean TP concentration of 2.76 mg L^-1^, which was the highest among all the treatments. The next highest concentration was from the mineral P (MinP, 1.50 mg L^-1^) and then incorporation of pig slurry (SP-Incor, 1.39 mg L^-1^) treatments (Table 5). The latter two treatments had significantly lower concentrations of TP in the leachate compared with SP-Surf, but there was no significant difference between them. Moreover, the treatments with P applications had different fractionations of P in the leachate. The proportion of DRP of TP ranged from 46% in the SP-Incor treatment to 77% in the MinP treatment.

Concentrations of DRP followed the same trend as TP: SP-Surf > MinP > SP-Incor > control, but without a significant difference between SP-Surf and MinP. Concentrations of other-P showed a different trend. The MinP treatment had a significantly lower concentration of other-P than the two slurry treatments, with no significant difference between the latter two.

In contrast to what was observed for the clay loam, P applications of 30 kg ha^-1^, to the loamy sand columns did not increase P leaching (Table 6). No significant difference in P concentrations among treatments was observed except that the concentration of other-P in the SP-Incor treatment was higher than that in the MinP treatment (*p* = 0.005). Concentrations of TP and DRP in the leachate from the loamy sand columns which received pig slurry or mineral P were even lower than the control (not significant), due to a relatively higher baseline P leaching from the latter (Table 5). Also, soil test P was higher in the loamy sand than the clay loam. Decrease in the concentrations of P in the control treatment indicated the existence of a dilution effect due to the large water input.

### Dynamics of P leaching

**Figure 1:**
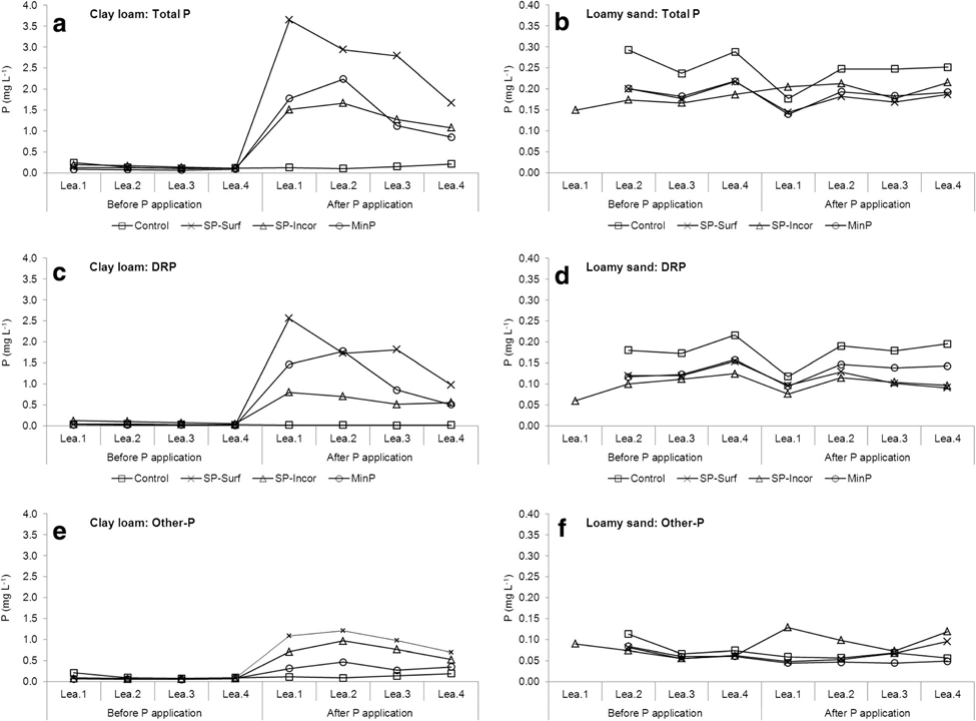
**Mean concentrations of total P, DRP, and other-P in each effluent sample before and after slurry or mineral P application to the soil columns (n=3 for the control of the clay loam throughout the experiment, and for the SP-Incor and MinP treatments of the clay loam after the laboratory P application, respectively; n=4 for all the other treatments).a**. Total P for the clay loam; **b**. Total P for the loamy sand; **c**. DRP for the clay loam; **d**. DRP for the loamy sand; **e**. Other-P for the clay loam; and **f**. Other-P for the loamy sand (Note that the scales of Y-axis differed with soils).

Figure 1 shows the concentrations of TP, DRP and other-P in each effluent sample from the two soils and four treatments. The concentrations of TP in each effluent sample from the clay loam columns increased dramatically after the columns received P compared with before application, which were low at each event with little variations. The concentrations of TP from the three treatments with P applications reached peaks of 3.65 mg L^-1^ during the first event (SP-Surf), and 2.24 mg L^-1^ (MinP) and 1.67 mg L^-1^ (SP-Incor) during the second event. Following this, concentrations decreased until the fourth event, with higher rates of decline in the treatments with surface-applied pig slurry or mineral P than the one with pig slurry being incorporated. Concentrations of TP, DRP and other-P in the SP-Surf treatment at each leaching event were higher than or equal to (only at one event, Figure 1c) those in MinP and SP-Incor treatments. The MinP treatment had higher concentrations of TP than the SP-Incor treatment during the first two leaching events, and lower during the last two, but with overall similar concentrations. The MinP treatment had higher or equal concentrations of DRP compared to the SP-Incor treatment at each event; while this was opposite for other-P.

Concentrations of TP, DRP and other-P from the columns with loamy sand were generally low for each event during the study, without significant increase after P applications to the columns. The SP-Incor treatment had the highest concentration of other-P at the first event, which was probably mainly PP created by disturbance of the soil surface when incorporating the slurry.

## Discussion

The overall good evenness of leachate amounts from the different treatments after applications of P enabled a relevant comparison between concentrations and leaching loads of P in the different treatments. One exception was the SP-Incor treatment of the loamy sand which had a slightly lower amount of leachate than the other treatments. Irrigation with tap water that had higher amounts of basic cations and ionic strength than natural rain water might have resulted in less transport of DRP (Jensen et al., 1998; Schärer et al. 2006) and colloidal P (Kaplan et al. 1996; Kretzschmar et al. 1999) than under field conditions. However, this was not likely to substantially affect relative comparisons between the soils and between the treatments in this study.

The differences in soil properties are the main reasons for different behaviors of P leaching from the two soils and responses to applications of P. A significantly higher baseline leaching of TP from the loamy sand than that from the clay loam was attributed to a higher soil P-AL value, in combination with a higher active flow volume, which is negatively correlated with clay content (Glæsner et al. 2011). The concentrations of TP in leachate from the loamy sand were well comparable with those reported by Siemens et al. (2008) on a soil with similar texture and dispersible P of 4.1 mg kg^-1^. However, the concentrations from both soils were low compared with those from a soil located nearby the field where the soil columns used in this study were collected, with similar texture but with a high soil P status (P-AL: 220–280 mg kg^-1^) (Liu et al. 2012b).

High P sorption capacity of the loamy sand and good contact between water and soil matrix are critical for the retention of P, which was very clear from the results of this study. There was no increase in P leaching from the columns after applications of P with slurry or mineral P, despite the fact that 80 mm water was added which was approximately equivalent to one pore volume. For the loamy sand, sorption of P by the soil matrix during water percolation alone managed to play a crucial role in retention of P. This sorption was effective enough and therefore overshadowed the effect of additional sorption of P by incorporation, which did not show any differences in TP leaching compared with when P was surface-applied. Application of P at a moderate rate, i.e. 30 kg ha^-1^ in this study, did not seem to be a problem in elevating concentrations of P in drainage from the sandy soil. This is consistent with the findings in a lysimeter study by Sørensen and Rubæk (2012) who did not observe detectable P leaching from a loamy sand and sandy loam to which solid pig manure was incorporated in autumn, due to sufficient P sorption capacity and absence of macropore flow. Moreover, 15 years measurements of drainage from field plots did not show any effect of yearly applications of manure on P leaching from a sandy soil, due to the high P sorption capacity of the subsoil (Liu et al. 2012a). However, potential P leaching may greatly increase when the soil has a low sorption capacity (Weaver and Ritchie 1994; Elliott et al. 2002), a high added P surplus, which exists in many areas with intensive livestock production around the world (OECD 2008), or a high degree of saturation with P after long-term supply with animal manure (Liu et al. 2012b). In addition, incidental losses of P occurring during rainfall instantly after P application, which is not uncommon, should be considered (Withers et al. 2003). Therefore, large P inputs to every soil may always constitute some risk of P losses. In the long-term, balanced P input with harvested biomass will be the best management practice to maintain soil fertility without increasing the risk of P losses.

This study confirmed that soil P status and P sorption capacity must be combined with information about P transport pathways to assess the risk of P leaching. Even though the clay loam had a high P sorption capacity, which was similar to the loamy sand, P leaching significantly increased after application of P. Phosphorus transport pathways were not explicitly studied here, but previous studies have shown the presence of macropore flow and a much lower active pore volume in clay and loam soils compared to sandy soils (Bergström and Shirmohammadi 1999; Glæsner et al. 2011). We believe that the increase in P leaching after P applications was most likely due to macropore flow through the soil. The columns were collected in autumn before plowing, i.e. before root and earthworm channels were destroyed, and water flow via macropores was therefore likely to occur. Incorporation of slurry to the clay loam significantly reduced P leaching compared to surface-application. Concentrations of TP were reduced by 50%, with a major reduction of DRP by, on average, 64%. The reduction of P concentrations was probably due to two things: incorporation of slurry allowed sorption of labile P in the slurry to the soil matrix to occur and simultaneously broke the continuity of macropores through which various forms of P may be transported rapidly. Other studies have also reported that mixing P with the topsoil, or injecting slurry to the soil, efficiently reduced potential of P losses by leaching (Djodjic et al. 2002; Glæsner et al. 2011) and also in surface runoff (Kleinman et al. 2002; Uusi-Kämppä and Heinonen-Tanski 2008). The reduction of TP leaching losses increases with increasing disruption of macropores (Geohring et al. 2001). However, DRP leaching in the SP-Incor treatment was still significantly higher than that in the control. In addition, incorporation of slurry had limited efficacy in reducing leaching of other-P, which did not significantly differ from surface application of slurry. For field conditions, where the slurry would be incorporated to a depth of 10 or 15 cm, retention of P in soil would probably increase. However, the slurry can never be mixed with the soil as thoroughly as in the laboratory, incorporation of slurry to soil under wet conditions would probably still constitute moderate to high risk for P leaching.

Even though the slurry had much lower labile P content (50% NH_4_Cl-extractable) than superphosphate (> 95% water soluble), surface-applied slurry resulted in DRP concentrations that were 50% (not significant) and other-P concentrations that were 180% (significant) higher than the mineral P application for the clay loam. A similar finding, that higher P leached from application of liquid manure than from mineral fertilizers, was shown in a previous column study where a high rate of P (166 kg ha^-1^) was applied on a fine sandy loam (Tarkalson and Leytem 2009). One explanation could be that organic acids and other organic materials in the manure can increase the mobility of P in soil by competing with P for sorption sties and increasing solubilization of P compounds (Bolan et al. 1994; Eghball et al. 1996). However, Borggaard et al. (2005) demonstrated that sorption of P to Fe and Al oxides is much stronger than to the organic acids such as humic and fulvic acids. Therefore, the high concentrations of P, especially other-P (colloidal P, DOP and PP), observed after application of slurry were probably mainly derived from the slurry.

Incorporation of slurry in the clay loam caused the same magnitude of TP leaching as the mineral P application, but with different forms of P dominating in the leachate. Even though thorough mixing of slurry in the upper soil substantially reduced DRP concentrations in the leachate, the concentration of other-P forms remained significantly higher than that by surface application of mineral P. The difference of other-P in the two treatments was probably due to the same reasons as explained before. It is difficult to explain why the mineral P application to the clay loam significantly increased concentrations of other-P in the leachate compared to before application. The most probable reason is that soluble P bound to particles or colloids during transport through the soil profile or even during storage of water samples in the laboratory. Sharpley et al. (1981) reported that soluble P sorbs to soil materials during transport in runoff at both watershed and laboratory scales. Although this was observed in runoff, the explanation probably also applies to P transport through the profile of the clay loam in the present study, where internal erosion via macropore flow may occur (Øygarden et al. 1997). Sorption of soluble P to the soil colloids may in turn enhance transport of colloidal P due to a reduction of the surface charge of the soil colloids (Ilg et al. 2008).

## Conclusions

The main objective of this study was to make a risk assessment of P leaching associated with P applications for two different soil types. We examined a scenario with application of manure before intensive irrigations approximately corresponding to one pore volume. The results stressed the importance of considering soil texture and structure, and water flow characteristics in addition to information about soil P status and P sorption capacity of the soil to identify high and low risk areas for P losses. We conclude that a sandy soil with low P saturation can be considered as a low risk soil and that slurry application at a rate approximately in balance with P removal with harvested biomass do not pose a major risk of P leaching, even under relatively wet conditions. For the structured clay loam, with apparent fast water flow pathways through macropores, the risk of P leaching was significant after P application. When slurry was mixed into the soil, TP leaching was reduced by 50% and DRP by 64%. However, application of slurry or mineral P before intensive irrigation constituted an increased risk of P leaching from the clay loam soil, irrespective of application methods. Thus, structured soils like the one included in this study should be considered as high risk soils and applications of P on bare soils during wet periods, e.g. in autumn, should be followed by incorporation. To minimize P leaching losses from a clay soil associated with manure application, other management practices besides incorporation of manure to the soil should be used. This indicates avoiding applications during the autumn period in cold and wet regions, and adopting improved tillage and/or cropping systems which may reduce P leaching via macropore flow pathways.

## Authors' information

Mr. JL is a PhD candidate, Dr. HA is an associate professor, and Dr. LB is a professor and division head, all at the Division of Biogeophysics and Water Quality Management, Department of Soil and Environment, Swedish University of Agricultural Sciences, Uppsala, Sweden. Dr. AS is a professor at Department of Crop, Soil and Environmental Sciences, University of Arkansas, Fayetteville, Arkansas, United States.

## Abbreviations

AL: Extraction with ammonium lactate
DOP: Dissolved organic P
DPS: Degree of P saturation
DRP: Dissolved reactive P
ICP: Inductively Coupled Plasma
ISO: International Standard Organization
MinP: Mineral P
PP: Particulate-P
PSI: P sorption index
SP-Surf: Pig slurry surface applied
SP-Incor: Pig slurry incorporated
TP: Total P.
